# Editorial: Reading in the Digital Age: The Impact of Using Digital Devices on Children's Reading, Writing and Thinking Skills

**DOI:** 10.3389/fpsyg.2020.586118

**Published:** 2020-09-16

**Authors:** Wai Ting Siok, Kang Kwong Luke

**Affiliations:** ^1^Department of Linguistics, The University of Hong Kong, Pokfulam, Hong Kong; ^2^School of Social Sciences, Nanyang Technological University, Singapore, Singapore

**Keywords:** reading and writing, digital devices, language and cognition, typewriting, literacy development

In the digital age, face-to-face communication is increasingly being supplemented, and in some cases even replaced, by computer-mediated communication. We interact less with each other directly but more through social media and other electronic platforms. New words and symbols are invented and syntax is modified to accommodate these needs, and these spread quickly through the virtual media. In schools and at home, children are being exposed to electronic-learning since a young age and knowledge acquisition is based on not just printed books but also electronic sources and internet contents. They handwrite less but type more, read less printed but more digital books, spell less but use more speech recognition engines to convert voice to text. Impaired readers may benefit from the audiobooks, and likewise dysgraphic children may write more by typewriting. How do the ever increasing exposure to electronic devices and the overwhelmingly rich information available on the internet affect children's reading, writing, and thinking skills? This Research Topic aims at understanding how the use of electronic devises has changed children's reading, writing, and thinking skills.

We are pleased to present four articles that contribute to this Research Topic. Two of the research papers suggest that careful use of digital tools may help children on their literacy skills. The study by Kloos et al. compares the effect of an online reading program with the usual classroom instruction on the reading fluency of elementary-school children. They report a positive effect of the former on the latter and explain how online programs can enrich individual experience by providing skill-level appropriate lessons. In a different study, O'Brien et al. apply tablet-based literacy intervention to groups of bilingual children with special education needs. They argue that real-time data can be collected from the tablets during the intervention to track individual needs and thus tailor-made interventions can be provided to children in need.

But caution must be exercised when using these virtual techniques with very young children. Mayer et al. report that, although kindergarten children trained to handwrite letters and words using paper and pencil do not differ from those trained to write using keyboard in word reading and writing, the handwriting group have better performance in letter recognition and visuo-spatial skills. Importantly, children trained to handwrite using a stylus on a touchscreen perform less well in all literacy dimensions, possibly because the slippery surface of a touchscreen increases difficulty of motor control. Echoing the importance of handwriting on word recognition, Merritt et al. suggest that children should not be taught to handwrite following a strict stroke order but should be allowed to handwrite in a self-directed manner.

In summary, findings based on alphabetic languages seem to suggest that electronic-based learning, if used carefully and wisely, may generate positive learning outcomes particularly for children with special needs. This view needs to be more rigorously tested in future research using an extended sample covering a wider age range and including more languages. We look forward to more studies on this topic.

## Author Contributions

All authors listed have made a substantial, direct and intellectual contribution to the work, and approved it for publication.

## Conflict of Interest

The authors declare that the research was conducted in the absence of any commercial or financial relationships that could be construed as a potential conflict of interest.

